# The Use of Three-dimensional Printer Molds for Treatment of Vaginal Agenesis

**DOI:** 10.1055/s-0042-1760208

**Published:** 2022-12-29

**Authors:** Claudia Cristina Takano, Marair Gracio Ferreira Sartori

**Affiliations:** 1 Department of Gynecology, Escola Paulista de Medicina, Universidade Federal de São Paulo, São Paulo, SP, Brazil

Although rare, vaginal agenesis is a relevant condition for gynecologists, who must be familiar with its current treatment.

It results from agenesis of the Mullerian ducts, known as Mayer-Rokitansky-Kuster-Hauser Syndrome (MRKHS), and the incidence is 1:5000 women. In this congenital malformation, genetic alterations affect the development of Mullerian ducts during the embryonic period and there is complete absence or significant hypoplasia of the uterus and vagina, with normal development of the external genitalia and breasts.

More rarely, the absence of the uterus and vagina is identified in patients with 46, XY Disorders of Sex Development (DSD) in which the presence of anti-Mullerian hormone inhibits the formation of the Mullerian ducts. In complete androgen insensitivity (Morris syndrome), the absence of testosterone action on its receptors leads to female differentiation of the external genital organs, and the conversion of testosterone to estrogen in peripheral tissues leads to the development of breasts at puberty. The clinical picture is similar to that of Rokitansky Syndrome, and in most cases, this is the initial diagnosis. The gynecologist will differentiate one from the other; in some cases, the suspicion is based on the lack of pubic and axillary hair and/or the presence of palpable gonads in the inguinal canal, but is confirmed by elevated levels of testosterone and the karyotype. Treatment will be the same as that of Rokitansky's syndrome, except for the recommendation to evaluate the gonads, given the higher risk of developing gonadoblastoma. The current recommendation is to wait for the end of puberty to consider gonadectomy, so that secondary characteristics can develop without the need for hormone replacement therapy. 1

As soon as the diagnosis is confirmed, the treatment of vaginal agenesis involves the steps established by the American College of Obstetricians and Gynecologists (ACOG). It begins by informing and advising the patient and her family about the condition, options and timing of treatment, and explaining about sexual relationships and reproductive future. It also involves referrals to psychological support and encouraging participation in support groups. 1

The approach regarding the formation of the neovagina is well established. The time to perform it is decided by the woman, when she manifests the desire to start a sexual relationship and demonstrates maturity and motivation to understand and participate in treatment, which generally occurs at the end of adolescence. Individual aspects inherent to this decision must be considered, such as the family context, religion and sexual orientation.

Since 2006, the ACOG recommends that “Nonsurgical creation of the vagina is the appropriate first-line approach in most patients”. 2 This approach is based on a success rate greater than 90%, which is similar to surgery, although with unquestionably smaller morbidity and costs. 1 3

Dilation is performed by the patient at home, on a daily basis, after detailed guidance from the gynecologist and supervision and follow-up throughout the process. Monitoring with a specialized physiotherapist is always beneficial, and essential when hypertonicity of the pelvic floor muscles is identified.

In Brazil, it is difficult to acquire rigid dilators, which are made of resistant material such as polylactic acid, since they are not commercially available in the country. Adapted devices such as acrylic candles and silicone dilators are commonly used.

The use of Additive Manufacturing (AM) technology and the three-dimensional printing device (3D Printer) have shown great potential for contribution and innovation in the health area. The 3D printer can create an object through its digital design. The model is evaluated and recognized by the three-dimensional printing device (3D Printer) through Computer Aided Manufacturing (CAM), the software that performs the processes of reading, analysis and digital slicing. Additive manufacturing technology is based on the deposition of layers to build the physical object. 4

The high customization capacity and the possibility of creating prototypes quickly, as well as the production of objects with complex geometries, have enabled the use of this technology in the development of products in the medical field. Additive manufacturing is also an economically viable technology in the production of small batches of customized products compared to conventional methods, making it an interesting alternative in the production and research of customized products. 4

According to a study by Fernandes et al. 5 published in the current issue, the application of this technology in the production of dilators for vaginal agenesis proved to be effective, economically viable, accessible and reproducible. Therefore, dilators can be produced in a gynecological care service equipped with a 3D printer and a qualified professional, allowing women with vaginal agenesis to have access to the recommended treatment for their condition. In addition to women with agenesis, these molds can also be used in other conditions in which dilation may be necessary, such as strictures and shortening of the vagina after radiotherapy or surgery.

There is also the possibility of using it in the manufacture of other devices in Urogynecology, such as customized pessaries for the treatment of genital prolapse and urinary incontinence.

The use of 3D printing technology reveals the importance of combining knowledge in the field of technology and health, as it enables the development of products with direct impact on medical treatment, in addition to opening up promising perspectives in other areas of Gynecology.
